# Navigating the challenges of overactive bladder management in women

**DOI:** 10.1007/s00345-026-06471-5

**Published:** 2026-06-01

**Authors:** Andrea Braga, Howard B. Goldman, Anna Padoa, Maurizio Serati

**Affiliations:** 1 Department of Gynecology and Obstetrics, Ente Ospedaliero Cantonale – Beata Vergine Hospital, Mendrisio, Switzerland; 2 Faculty of Biomedical Sciences, Università della Svizzera Italiana, Lugano, Switzerland; 3 Glickman Urologic Institute, Lerner College of Medicine, Cleveland Clinic, 9500 Euclid Ave, Q10, Cleveland, OH 44195 USA; 4 Department of Obstetrics and Gynecology, Chaim Sheba Medical Center, Ramat Gan, Israel; 5 Faculty of Medical & Health Sciences, Tel-Aviv University, Tel Aviv, Israel; 6 Department of Obstetrics and Gynecology, Del Ponte Hospital, University of Insubria, Varese, Italy

**Keywords:** Adrenergic beta-3 receptor agonists, Muscarinic antagonists, Overactive bladder, Urgency incontinence, Women’s health

## Abstract

**Purpose:**

Overactive bladder (OAB) is common among women, increases in prevalence with age, and exerts a considerable physical, psychological and socioeconomic burden. This narrative review discusses issues specific to the management of OAB in women.

**Methods:**

The review is based on discussions at a symposium held at the International Urogynecological Association/European Urogynaecological Association meeting (Barcelona, Spain; June 2025) supplemented by a PubMed search of articles (published in English since 1 January 2020) describing current treatment practices, unmet clinical needs and future directions for the management of OAB in women.

**Results:**

The pathophysiology of OAB in women is multifactorial, involving detrusor overactivity, urothelial and sensory dysfunction, and central nervous system dysregulation, with additional contributions from hormonal and age-related changes. Management of OAB has evolved toward an individualised, stepwise approach. Behavioural and lifestyle interventions remain first-line therapy, followed by pharmacological options, such as antimuscarinics and β3-adrenergic agonists. For refractory cases, onabotulinumtoxin A and neuromodulation are established third-line treatments. Local oestrogen therapy or prasterone may provide benefit in selected postmenopausal women, whereas radiofrequency and urethral bulking strategies remain investigational. Emerging evidence supports newer β3-agonists, such as vibegron, which may provide comparable efficacy to established β3-agonists (e.g. mirabegron) with improved cardiovascular tolerability. Treatment selection for women with OAB should consider comorbidities, drug safety, cognitive and cardiovascular risk in older patients, and challenges with adherence.

**Conclusion:**

Further research into underlying mechanisms, long-term outcomes and strategies to optimise treatment adherence will be essential to improve symptom control and quality of life for women living with OAB.

## Introduction

The International Continence Society recognises overactive bladder (OAB) as being suggestive of lower urinary tract dysfunction, defining the symptom syndrome as “*urgency*,* with or without urge incontinence*,* usually with frequency and nocturia*” in the absence of urinary tract infection or other obvious pathology [1]. Urgency is defined as a sudden compelling desire to void that is difficult to defer, increased frequency of urination as more frequent urination than deemed normal (which can occur during the day or at night [nocturia]), and urgency incontinence as involuntary loss of urine associated with urgency [2]. OAB accompanied by urgency incontinence is termed OAB-wet and OAB without urgency incontinence is termed OAB-dry [3]. Given that OAB is a symptom-based syndrome, variations in symptoms exist between certain patient groups (e.g. men and women) that reflect differences in the underlying pathophysiology [4].

OAB is very common worldwide, although the prevalence is higher among women than in men [4, 5]. A recent meta-analysis estimated the overall global prevalence at 21.9% in women versus 16.1% in men (19.5% overall) [5]. In particular, OAB with urgency incontinence (i.e. OAB-wet) appears to be significantly more prevalent in women than in men [6]. The prevalence of OAB also increases with age [5]; a Finnish population-based study found the prevalence of OAB ranged from 5.9% in women aged 18–29 years to 8.5% in women aged 40–49 years and 15.6% in women aged 70–79 years [7, 8].

Often underappreciated, OAB can impose a significant burden on women’s daily activities, quality of life (QoL) and sexual health [4, 9–13]. In particular, urgency incontinence is associated with more bothersome symptoms and has a significantly greater effect on mental health and healthcare utilisation than other types of urinary incontinence [14]. Women with OAB report feelings of distress and desperation, embarrassment, isolation and stigma associated with the disease [15]. Socioeconomic impacts of OAB include higher rates of unemployment and increased impairment of work productivity when compared with matched controls without OAB [9, 16]. Despite the negative impact of this syndrome, many women with OAB never seek medical help and/or remain untreated [17, 18]. The reasons for this may include a lack of awareness about effective management options, fear of drug adverse effects or the cost of treatment [19].

In this narrative review, we discuss the current state of OAB management in women in the hopes of improving patient care in the future.

## Evidence acquisition

The review is based on the topics raised and discussions during a Pierre Fabre-sponsored symposium at the International Urogynecological Association (IUGA)/European Urogynaecological Association (EUGA) joint congress held in Barcelona, Spain (18–21 June 2025).

A supplementary literature search was also conducted after the symposium to ensure that all key evidence related to these topics was adequately captured. A PubMed search was conducted on 26 August 2025 to identify published articles that describe current treatment practices, unmet clinical needs and future directions for the management of OAB. The initial search was performed by a medical writer at Springer Health+. Key search terms included “*overactive bladder (Title)*” in combination with Title/Abstract terms relevant to the topics discussed during the IUGA/EUGA symposium (e.g. “*definition*”, “*pathophysiology*”, “*burden*”, “*treatment*”, “*menopause*”, “*adherence*” and “*elderly*”). The search was limited to English-language papers published since 1 January 2020 to ensure consideration of the most recent evidence. The titles and abstracts of the search results were screened to identify key publications that supplemented the evidence presented at the IUGA/EUGA symposium. Additional relevant information was identified from the reference lists of the articles selected from the literature search, and from the authors’ knowledge of the area.

### Overactive bladder: pathophysiology in women

The pathophysiology of OAB in women is complex and multifactorial, involving abnormalities in bladder function, urothelial signalling, sensory pathways and central nervous system (CNS) regulation [4]. Normal bladder function involves relaxation of the bladder during the storage phase and detrusor contraction/urethral sphincter relaxation (coordinated by the CNS) during voiding [20]. Balance between the storage and voiding phases is neurologically regulated by peripheral inputs from bladder and urethral sensory nerves, and central modulation involving the brainstem, cortex and spinal cord [21]. Proposed mechanisms of OAB include detrusor overactivity resulting from spontaneous or involuntary detrusor contractions during bladder filling; hypersensitivity of the bladder wall caused by increased afferent signalling, even when not full; structural or functional alterations in the urothelium/suburothelium; and brain–bladder axis dysfunction [4].

Comorbidities and risk factors for OAB include pelvic floor dysfunction and childbirth-related changes [22–24]; neurological disease (stroke, multiple sclerosis [MS], Parkinson’s disease) [4, 24]; and metabolic syndrome, obesity and diabetes with associated inflammation and neuropathy, which can contribute to bladder hypersensitivity [4, 5, 12, 22, 24, 25].

Obstetric events increase the risk of pelvic floor disorders and urinary incontinence. Vaginal delivery appears to be a particular risk factor for these disorders; a large (*n* = 1481) prospective, longitudinal study from the United States (US) reported the prevalence of highly bothersome OAB during the first 5 years after delivery to be over 4-fold higher in women who had given birth vaginally than in those who delivered by caesarean Sect. (6.0% vs. 1.4%, respectively) [26].

Pelvic organ prolapse is associated with OAB [27–30], with studies reporting symptoms of OAB in 80% of women with a prolapse [29] and prolapse in 70–90% of women with symptoms of OAB [28, 30]. The location of the prolapse appears to influence symptom severity, with a study of Finnish women (*n* = 2933) finding increased urinary frequency with advancing anterior wall and apical prolapse, and increased severity of urgency urinary incontinence with advancing anterior wall prolapse [29].

Over 80% of patients with MS report symptoms of lower urinary tract dysfunction, with those related to the storage phase the most frequent [31]. Female sex has been reported to be one of the variables (in addition to more severe disability and lack of disease-modifying treatments) associated with urinary incontinence in patients with MS [32].

Bladder dysfunction is a common symptom caused by autonomic impairment in Parkinson’s disease [33, 34], with the risk of OAB being significantly higher in patients with Parkinson’s disease than in healthy individuals [35]. A survey-based study of women with Parkinsons’s disease found they had significantly higher self-reported urinary urgency or incontinence than age-matched controls (68% vs. 43%, respectively) [36].

Symptoms of urinary incontinence occur in up to 80% of individuals who have experienced a stroke [37]. A large population-based study of Korean women reported that stroke (in addition to other variables such as advanced age, high body mass index [BMI], and a history of hypertension, diabetes and hyperlipemia) was significantly associated with OAB [38]. A study of Japanese patients with lower urinary tract symptoms (LUTS) following a stroke found that female (versus male) sex was significantly associated with OAB symptoms of urgency, urgency incontinence and stress incontinence, but not other symptoms such as daytime frequency and nocturia [39].

Obesity is strongly associated with OAB. A study of women of varying weight (normal weight, overweight and obese) found obesity to be an independent predictor for OAB and detrusor overactivity [40]. Interestingly, other components of the metabolic syndrome (diabetes, hypertension and dyslipidaemia) were not associated with OAB in this study [40]. These findings contrast those of a meta-analysis of women with OAB that found those with the disorder had significantly higher BMI and waist circumference but also significantly higher fasting glucose, triglyceride and LDL-cholesterol levels, and lower HDL-cholesterol levels than a control group without OAB [41]. Another study found the occurrence of both OAB-wet and OAB-dry to be significantly higher in obese women than women with a normal BMI [22]. Body fat percentage (BFP) may be a driver of these associations, with a study reporting a prevalence of OAB of 58% in women with a BFP > 32% versus only 12.2% in women with BFP of < 32% [25]. Obesity is associated with excessive adipose tissue in the abdominal cavity (central obesity), which may increases intra-abdominal pressure and affect the bladder and stretch the pelvic floor [42]. A large population-based study from the US found a positive association between waist circumference and OAB prevalence in women, and that higher circumference was associated with an increased risk of OAB [43]. Women appear to be more susceptible to the impact of obesity on OAB, given evidence that the relationship between waist circumference and OAB exists in women but not in men [42].

Mental health disorders (specifically, depression, anxiety and post-traumatic stress disorder) have been reported to be associated with OAB and may represent risk factors for the development of the condition [44, 45]. There is evidence that anxiety may impact hypersensitivity mechanisms that may underlie and contribute to the development of OAB in some women [46].

## Hormonal and age-related changes in the development of OAB in women

In women, the pathophysiology of OAB is further influenced by hormonal and age-related changes. The presence of oestrogen receptors (ERs) throughout the lower urinary tract is consistent with the likelihood that oestrogen modulates urinary tract functions [47]. ER-beta predominates in the bladder and urethra [47]. ER expression in the bladder and urethra is similar among women in different oestrogenic states, suggesting that the female lower urinary tract is receptive to the actions of endogenous and exogenous oestrogens [47]. Low oestrogen levels cause amplification of acetylcholine release in the bladder, accompanied by increased urinary frequency and decreased voided volume [47]. This increased acetylcholine release can be reversed by exogenous oestrogen replacement [47]. 17β-oestradiol has been shown to reduce spontaneous phasic detrusor contractions through direct activation of big potassium (BK) channels, the predominant potassium channels in the bladder that regulate smooth muscle function [47].

The term ‘genitourinary syndrome of menopause’ (GSM) has been introduced to describe the hormonal impact of menopause on the genitourinary system. GSM may overlap with OAB through both hypo-oestrogenic changes and microbiome alterations [48]. Clinical studies investigating the effects of oestrogen decrease on LUTS have shown changes occurring during menopausal-associated decreases in oestrogen levels, such as urethral shortening, thinning of the urethral mucosa, decreased urinary sphincter contractility and reduced bladder compliance [47]. Studies have also reported a spike in the prevalence of urinary incontinence around the time of menopause, with the onset of urinary incontinence in women often associated with the final menstrual period [47]. Moreover, urodynamic evaluation of menopausal women with urinary incontinence has demonstrated inhibition of filling phase bladder contractions and increased urethral closure pressure with local oestrogen therapy, possibly due to effects on the urethral vascular network [47].

## Current management of OAB in women

**Fig. 1:**
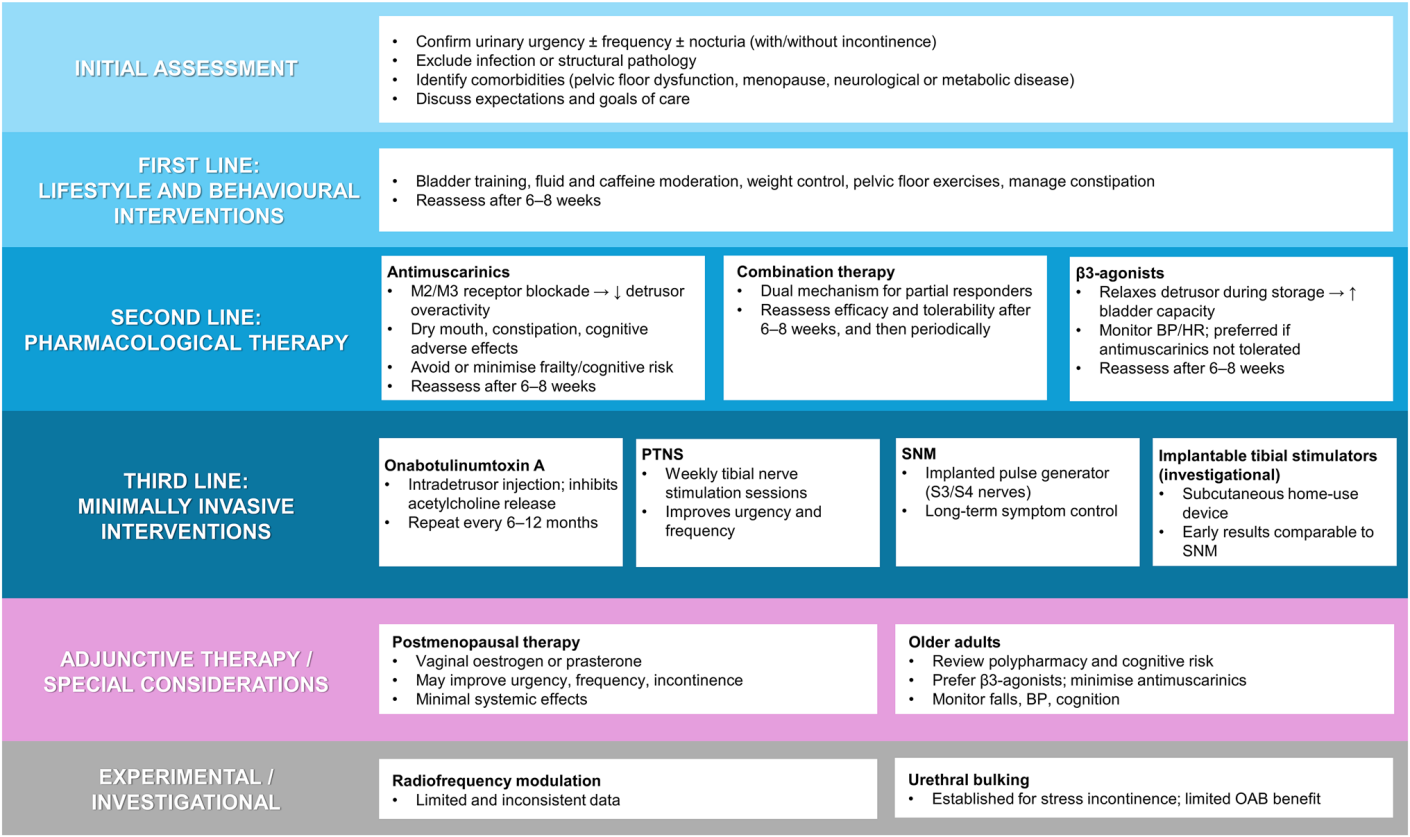
Management algorithm for women with OAB, including considerations for older adults. *BP* blood pressure, *HR* heart rate, *OAB* overactive bladder, *PTNS* percutaneous tibial nerve stimulation, *SNM* sacral neuromodulation

A summary of current management approaches for women with OAB is provided in Fig. 1.

First-line management of OAB is mainly composed of lifestyle interventions (e.g. bladder training, pelvic floor muscle training, dietary modifications, physical activity/exercise, weight loss, reduced intake of fluid/caffeine/alcohol) [24, 49–52]. Cognitive-behavioural therapy (CBT) may also have a place in management. According to a systematic review of the effectiveness of standalone CBT in women with urinary incontinence or stress urinary incontinence with predominant urge urinary incontinence and/or OAB, CBT was effective in reducing symptom severity, and possibly improving on QoL, psychological symptoms and patient satisfaction, but there was no evidence of effects on clinical signs (i.e., urodynamic testing, pelvic floor muscle strength) [53]. Patients should be counselled about the need for long-term (lifelong) compliance to maintain the effect of non-invasive therapies (such as bladder and pelvic floor muscle training) prior to initiating these approaches [50]. While patients may self-medicate with over-the-counter products (such as nutraceuticals, vitamins, supplements or herbal remedies), the guidelines from the American Urological Association (AUA) state that there is currently insufficient evidence to support the use of these products to manage OAB [50].

Recommended second-line management includes the use of antimuscarinic drugs and β3-agonists (Table 1) [24, 49–51].

**Table 1:** Established pharmacotherapy options for women with OAB (see text for references)

Class/agent	Efficacy	Typical AEs	Cognitive effects	Falls/frailty	CV effects	Persistence/adherence	Notes
Antimuscarinics							
Oxybutynin	Class-comparable	Class-typical anticholinergic effects	Short-term impairment shown; class association with dementia in observational studies	Falls risk greater with oxybutynin than with β3-receptor agonists	Antimuscarinic class signal includes ↑HR/QT prolongation; solifenacin cases specifically reported; agent signals vary	Lower 12-month persistence than β3-agonists	Associated with higher all-cause mortality vs. other OAB medications in observational studies; consider alternatives in older / frail women
Solifenacin	Class-comparable	Class-typical anticholinergic effects	Class association with dementia in observational studies	Anticholinergic burden contributes to falls risk	QT prolongation cases reported; CV effects appear more common with solifenacin than others	Moderate; discontinuation often AE/expectation-driven	Use lowest effective dose in at-risk women
Fesoterodine	Class-comparable	Class-typical anticholinergic effects	Class association with dementia in observational studies	Anticholinergic load → falls risk	No specific QT signal	Moderate	Balance efficacy vs. AEs
Darifenacin	Class-comparable	Class-typical anticholinergic effects	Class association with dementia in observational studies	Anticholinergic load → falls risk	No specific QT signal	Moderate	Use cautiously in older women
Trospium	Class-comparable	Class-typical anticholinergic effects	Class association with dementia in observational studies	Anticholinergic load → falls risk	No specific CV signal	Moderate	Dose-adjust in renal impairment as per local labelling
Tolterodine	Class-comparable	Class-typical anticholinergic effects	Class association with dementia in observational studies	Anticholinergic load → falls risk	No specific QT signal	Often limited by AEs/expectations	Access varies by formulary
β3-adrenergic agonists							
Mirabegron	Similar reductions in urgency/UUI to antimuscarinics in trials/real-world studies	Fewer anticholinergic AEs vs. antimuscarinics	Non-anticholinergic	Non-anticholinergic	Modest ↑BP/HR (< 5 bpm); no QT prolongation signal; monitor BP/HR	Generally higher than antimuscarinics	Cost/coverage may influence choice
Vibegron	Similar to mirabegron/anti-muscarinics	Fewer anticholinergic AEs vs. antimuscarinics	Non-anticholinergic	Non-anticholinergic	Trial data suggest BP/HR neutrality; higher β3-receptor selectivity	Real-world persistence greater than mirabegron (65.2% vs. 30.3%)	Cost/coverage variable; emerging availability

*AE* adverse event, *BP* blood pressure, *bpm* beats per minute, *CV* cardiovascular, *HR* heart rate, *OAB* overactive bladder, *UUI* urgency urinary incontinence, *vs.* versus

Antimuscarinic drugs (oxybutynin, tolterodine, solifenacin, fesoterodine, darifenacin, trospium) block M2/M3 muscarinic receptors in the detrusor muscle [54]. Among the antimuscarinics, no particular agent has shown clear superiority over the others, and patients who fail to respond to a first-line antimuscarinic therapy generally do not respond to higher doses or another antimuscarinic agent [51, 55, 56]. There is some evidence that the combination of an antimuscarinic and desmopressin may be effective for managing OAB in women [57] and that the combination of an antimuscarinic with the serotonin-noradrenaline reuptake inhibitor duloxetine can improve symptoms of urge-predominant mixed urinary incontinence in women [58]. Inconsistent evidence is available regarding the efficacy of a combination of an antimuscarinic and local oestrogen, with one study finding no added benefit of oestrogen [59] but two others reporting greater improvements in some but not all OAB symptoms and improved sexual function and QoL with the combination versus an antimuscarinic alone [60, 61]. Antimuscarinic-related adverse effects include anticholinergic effects, such as dry mouth, constipation, dizziness, voiding difficulties and cognitive impairment [54].

β3-agonists (e.g. mirabegron) are now established as first-line pharmacological alternatives to antimuscarinics in international society guidelines, such as those of the AUA and the European Association of Urology (EAU) [50, 51]. These agents work by relaxing the detrusor muscle during storage, increasing bladder capacity [54]. They can be used as monotherapy or in combination with antimuscarinics for additive efficacy in patients with refractory symptoms [50, 51, 62], and are preferred in patients who are intolerant of antimuscarinics or at risk of anticholinergic cognitive adverse effects [54, 56]. However, mirabegron is associated with hypertension and increased heart rate [54].

In clinical practice, the choice of treatment between these two drug classes with distinct mechanisms of action is influenced by their differing contraindications, tolerability profiles and practical considerations, such as cost and insurance coverage [54, 63, 64]. In particular, careful consideration of possible adverse events is critical in determining the first and sequential choice of treatments [54].

Treatment options for refractory OAB involve third-line minimally invasive interventions, such as onabotulinumtoxin A and electric neuromodulation [24, 49, 50, 65]. Onabotulinumtoxin A injections administered via cystoscopy into the bladder wall inhibit acetylcholine release, thereby reducing detrusor overactivity [24, 51, 65, 66]. Repeated treatments every 6–12 months are usually required [24, 65]. Percutaneous tibial nerve stimulation (i.e. stimulation of sacral afferents via the tibial nerve administered as weekly outpatient sessions) and sacral neuromodulation (i.e. an implanted device stimulating sacral nerves) directly or indirectly influence the function of the afferent nerves in the bladder to restore imbalanced stimulating/inhibitory control systems [51, 65, 66]. Implantable tibial nerve stimulation devices placed subcutaneously near the ankle have also become available in some regions. Early prospective studies and an indirect meta-analysis have reported OAB and urgency incontinence responder rates comparable with sacral neuromodulation, with low rates of device-related adverse events, although follow-up remains limited and head-to-head comparative data are not yet available [67, 68].

In addition, vaginal oestrogen therapy has demonstrated improved urinary frequency, urgency and incontinence in postmenopausal women with OAB [69–74]. The efficacy of vaginal oestrogen has been reported to be similar to that of tolterodine in women with OAB [75]. Prasterone, a synthetic form of dehydroepiandrosterone approved for the treatment of dyspareunia and moderate-to severe vulvovaginal atrophy, has also demonstrated improvements in urgency, urinary incontinence, nocturia and frequency when administered intravaginally in women with OAB [76–79]. Although the evidence remains limited to small and mostly uncontrolled studies, the symptomatic overlap between GSM and OAB supports consideration of a short trial of topical oestrogen or prasterone in postmenopausal women, given the favourable local tolerability and safety profiles of these drugs.

## Current challenges of pharmacotherapy for OAB management in women

The limitations of antimuscarinic drugs include modest efficacy and poor tolerability (dry mouth, constipation, urinary retention, blurred vision, cognitive impairment), which often lead to high rates of treatment discontinuation [80]. The limitations of β3-agonists are that long-term cardiovascular safety continues to be monitored but is not confirmed, their higher cost relative to antimuscarinics can limit access, and their mechanism of action does not address all pathophysiological drivers of OAB (e.g. CNS contributions) [80]. Some of these challenges are discussed in more detail below.

### Medication adherence and persistence

OAB is a chronic condition, with one report finding that symptoms lasted ≥ 10 years in the majority (88%) of women in the sample [81]. However, another study found that the disease is dynamic and characterised by periods of stability, remission and progression [82]. Given the chronicity of OAB, adherence to treatment regimens is imperative to ensure successful management. Patients should be counselled about the chronic nature of OAB and, thus, the need for long-term treatment and strict adherence to their prescribed medication(s).

However, despite greater persistence with antimuscarinics being associated with better health-related QoL [83], 12-month persistence rates for OAB medications are poor, ranging from 5% to 68% [84–89]. Reported 24-month persistence rates range from 49% to 84% [85, 89, 90]. A retrospective cohort study that investigated persistence with treatments for OAB specifically in women found persistence rates of 55%, 46% and 37% over 30 days, 90 days and 1 year, respectively [86].

Lower rates of persistence and adherence have been reported in younger versus older patients [83, 85, 88, 89, 91]. Data regarding a difference in persistence and adherence to OAB treatments by sex are inconsistent, with studies finding higher [91] and lower [88] rates of persistence in females than in males. Persistence appears to be better with β3-agonists than with antimuscarinics [84, 92], with specific data indicating that this advantage for β3-agonists is observed in women [86, 93].

A primary reason for discontinuing OAB medication is a lack of efficacy; this may be driven by the unrealistic expectations of some patients [87, 88, 94–97]. Other common reasons for discontinuing OAB medications are adverse events and the cost of treatment [87, 88, 94–97]. There are limited and conflicting data to suggest that switching from one antimuscarinic drug to another leads to improved persistence [88, 90].

### Cognitive impairment and dementia

Treatment of OAB can be particularly challenging in older versus younger women. Older individuals are at risk of polypharmacy with other anticholinergic drugs (e.g. antidepressants and anti-Parkinson, antipsychotic and antiepileptic drugs) [98]. Clinical studies of antimuscarinics have reported an increased risk of developing cognitive impairment/dementia [98–106]. Loss of cognitive function is of particular concern to older people receiving treatment for OAB [107].

Cholinergic signalling is ubiquitous throughout the brain [105]. M1 receptors are more abundant than M2 and M4 receptors, being highly expressed in the hippocampus and neocortex [101, 105]. Cholinergic neurones play a critical role in memory, learning and attention [105]. Short-term clinical studies have shown oxybutynin use is associated with impaired memory (comparable with up to 10 years of normal aging), attention, reaction time and sleep structure [102, 104, 108, 109]. Short-term clinical studies of other antimuscarinics in OAB (i.e. darifenacin, solifenacin, fesoterodine, trospium) showed no cognitive impairment, likely as a result of varying ability of the drugs to cross the blood–brain barrier [102, 104, 105, 109, 110]. However, large observational studies and a 2020 meta-analysis indicated that antimuscarinics may be associated with an increased risk of dementia [98, 111, 112].

Despite clinical evidence supporting an association between antimuscarinics and dementia, this may not mean causation. OAB and urgency incontinence are more prevalent in Alzheimer’s disease, with LUTS correlating with disease severity [113, 114]. An Alzheimer’s mouse model showed changes in bladder innervation that may lead to functional bladder changes [115]. However, a large, Canadian, population-based, propensity-matched cohort study suggested a causative link, with an increased risk of new-onset dementia observed among patients with OAB who were treated with antimuscarinics versus β3-agonists [116]. Interestingly, sex was a significant effect modifier, with a significantly lower risk of dementia from antimuscarinics in women than in men [116].

### Falls and fractures

Falls are the leading cause of unintentional injury in people aged > 65 years [117, 118], with 87% of all fractures in older individuals being caused by falls [119]. Falls are associated with mortality and hospitalisation [117, 119, 120], accounting for 32% of moderate-to-severe injury requiring hospital admission [121]. The risk of falling increases with age and antimuscarinic burden [120, 122]. Other risk factors include balance and gait disorder, polypharmacy, female sex and visual impairment [118, 120, 123, 124].

OAB presents a risk of falls [125], due to factors such as the diverting of cognitive attention in the midst of urgency [126] and the need to get out of bed to urinate during the night [127]. Indeed, a Japanese study of women with OAB found that nocturia (and more severe OAB) had the strongest correlation with fracture risk [123]. Female sex has been identified as a risk factor for fall-induced bruising in individuals with OAB symptoms [118]. A Canadian retrospective cohort study of mostly females found that the risk of falls in individuals with OAB was higher with oxybutynin versus β3-agonist treatment [128], and female sex was identified as a risk factor for falls in a Germany study of patients taking antimuscarinic agents for OAB [120].

### Cardiovascular considerations

Among recommended pharmacotherapies, both antimuscarinics and β3-agonists are associated with cardiovascular effects. The most common cardiovascular adverse events with antimuscarinics are increased heart rate and QT interval prolongation [129, 130]. Three cases of serious QT prolongation and polymorphic VT (TdP) have been reported with solifenacin [129, 131]. Of note, cardiovascular adverse effects appear to be more common with solifenacin than with other antimuscarinics [129, 131]. While the β3-agonist mirabegron does not appear to cause QT prolongation at therapeutic doses [132], it is associated with modest hypertension and increased heart rate (< 5 bpm) [129, 132–136].

Thus, consideration should be given to the potential for adverse effects on cardiovascular function when prescribing OAB therapies particularly in older individuals with cardiovascular risk factors and comorbidities [129]. Two issues in this regard are pertinent for women. First, the risk of hypertension, dyslipidaemia and cardiovascular disease increases markedly after menopause, as a result of a reduction in the levels of oestrogen, a cardioprotective hormone [137]. Thus, the potential effect of OAB medications on cardiovascular parameters should be considered when prescribing them to women with OAB, particularly to those who are post-menopausal. Second, female sex has been reported to be a risk factor for drug-induced torsade de points [138] and, therefore, the risk of QT interval prolongation should be considered when prescribing OAB medications to women.

### Mortality risk

Oxybutynin is associated with a higher risk of all-cause mortality compared with other OAB antimuscarinic medications and β3-agonists [130]. A retrospective cohort study showed that nonselective antimuscarinics had a 50% higher risk of 180-day mortality compared with selective antimuscarinics in patients with dementia and OAB [139].

### Management of OAB in older women

Traditionally, OAB management has involved the use of first-line behavioural therapy and pelvic floor physiotherapy, second-line pharmacological agents and third-line minimally invasive interventions [24]; however, the most recent guidelines from the AUA and the Society for Urodynamics, Female Pelvic Medicine, and Urogenital Reconstruction (SUFU) are less linear and more individualised [50]. In older patients with OAB, initial treatments should be those with the least potential for harm [24].

It is important to recognise that older individuals have a higher prevalence of comorbidities and polypharmacy, thus are more likely to be receiving multiple anticholinergic drugs [140]. Older patients may also be more susceptible to off-target anticholinergic effects resulting from an age-related decline in cholinergic function, increased blood–brain barrier permeability and altered drug pharmacokinetics [140].

A SUFU white paper on OAB antimuscarinics and dementia risk recommended that potential harms should be balanced against potential improvement in QoL with treatment [106]. Other organisations recommend minimising the number of antimuscarinics and for a β3-agonist to be used first [50, 141].

The American Urogynecologic Society (AUGS) consensus statement for treating OAB in women recommends using the lowest effective dose of all antimuscarinic medications to lower the overall anticholinergic burden and to consider alternative medications, such as β3-agonists [142]. The AUGS and the Pelvic Floor Disorders Research Foundation also suggest that non-antimuscarinic therapies be emphasised in the older population with cognitive impairment [100]. The AUA/SUFU guidelines recommend maximising non-pharmacological management and discontinuing antimuscarinic therapy if it is ineffective [50]. It is typical for women with OAB to try multiple treatments before symptom relief is considered satisfactory [143]. Indeed, the AUA/SUFU guidelines state that a medical history and comprehensive assessment of symptoms should be conducted before prescribing pharmacotherapy, with caution exercised in the older/frail population and treatment coordinated with the individual’s general practitioner [50]. The potential risk of cognitive impairment and dementia should also be discussed with individuals who are prescribed antimuscarinics [50].

### Management of OAB in pregnant women

In general, the use of OAB medications is discouraged during pregnancy, as OAB anticholinergic medications have been associated with a higher risk of pregnancy complications [144]. While data are limited, the results of this study suggest that the first-line management of OAB in pregnant women should prioritize non-pharmacological methods (behavioural therapies).

## Future directions for the management of OAB in women

### Vibegron

Vibegron is a second-generation β3-agonist that has shown efficacy and safety for the treatment of OAB in randomised clinical trials [145] and in specific analyses of female study participants [146, 147]. Vibegron was approved by the US Food and Drug Administration in 2020 [148]. It is more selective for β3 receptors than mirabegron, with negligible β1 or β2 receptor activity [149]. β3 receptors are predominantly expressed in the bladder and detrusor smooth muscle, accounting for approximately 95% of β-adrenergic receptors, which aid detrusor smooth muscle relaxation during bladder filling [149]. However, human β3-adrenergic receptors share approximately 50% of their sequence with β1- and β2-receptors, and since β-adrenergic receptors are also expressed in cardiovascular tissue, off-target binding can result in cardiovascular adverse effects [149, 150]. The functional effects of β3-agonists include reduced detrusor contractions during bladder filling and increasing storage capacity without impairing voiding [151].

Vibegron has efficacy similar to that of mirabegron in reducing urinary urgency and incontinence episodes [147, 152]. A study of Japanese women reported no significant difference in efficacy (daytime frequency, nocturia, urgency and urinary urgency with incontinence) between the two treatments, although there was a significantly higher incidence of treatment-related adverse events in the vibegron group (38.5% vs. 19.1%, respectively) [147]. The high β3 selectivity of vibegron results in fewer cardiovascular adverse effects than mirabegron, with no apparent effects on blood pressure or heart rate [147, 150, 153]. Vibegron has shown good adherence in the real-world setting, and persistence with vibegron is greater than with mirabegron (65.2% vs. 30.3%) [154, 155]. Furthermore, vibegron has been associated with statistically significant and clinically meaningful improvements in QoL compared with placebo [156], and appears to be cost-effective relative to antimuscarinic OAB drugs [157, 158].

### Radiofrequency

Radiofrequency-based therapies (i.e. transvaginal detrusor nerve ablation and fractional microablative and transcutaneous temperature-controlled radiofrequency) have been explored as potential neuromodulatory approaches for OAB in women, targeting autonomic nerves and ganglia in the bladder outlet and trigone [159–164]. Although small studies have reported symptomatic improvements with few adverse effects [160, 163, 164], the evidence remains limited and inconsistent, and radiofrequency-based therapies are not currently used in routine clinical practice.

### Urethral bulking agents

Urethral bulking agents are injectable materials (e.g. water-soluble gel, silicone particles, collagen, carbon-coated beads) that are deposited into the urethral submucosa to bulk the urethral wall, narrow the lumen, improve urethral closure and prevent urinary leakage [165, 166]. Data on the use of urethral bulking agents for the treatment of stress urinary incontinence in women appear promising [165, 167]. Limited data from observational studies in women with mixed urinary incontinence (urodynamic stress urinary incontinence with detrusor overactivity) suggest that these agents may be useful therapeutic options in some individuals with OAB. However, it should be noted that these data are from non-randomised studies and agent-specific [167]. Furthermore, urethral bulking agents are an established treatment for stress urinary incontinence rather than OAB.

## Conclusions

OAB in women is a multifactorial disorder with complex underlying mechanisms and a significant impact on QoL. Current management is becoming increasingly individualised, following a stepwise approach that balances efficacy, safety, tolerability and patient preference. Behavioural and lifestyle interventions, antimuscarinics and β3-agonists remain the standard-of-care treatment, while onabotulinumtoxin A, neuromodulation and newer agents (e.g. vibegron) are emerging available options. Local hormonal therapies may benefit selected postmenopausal women, whereas radiofrequency and urethral bulking techniques remain investigational. Further research into disease mechanisms, long-term safety and real-world treatment persistence will be essential to optimise outcomes and improve care for women with OAB.

## Data Availability

No datasets were generated or analysed during the current study.

## Material/code availability

Not appliable.
